# Genetic Diversity and Clonal Expansion of Pathogenic *Leptospira* in Brazil: A Multi-Host and Multi-Regional Panorama

**DOI:** 10.3390/microorganisms13112512

**Published:** 2025-10-31

**Authors:** Maria Isabel Nogueira Di Azevedo, Walter Lilenbaum

**Affiliations:** 1 Laboratory of Veterinary Bacteriology, Biomedical Institute of Fluminense, Federal University, Niteroi 24230-340, Brazil; wlilenbaum@id.uff.br; 2 Laboratory of Investigation in Medical Microbiology, Institute of Microbiology, Federal University of Rio de Janeiro, Rio de Janeiro 21941-902, Brazil

**Keywords:** molecular epidemiology, phylogenetics, haplotype network, zoonosis, one health

## Abstract

Leptospirosis is a globally distributed zoonosis of major public health and veterinary relevance, caused by pathogenic species of the genus *Leptospira*. Brazil is a hotspot for transmission due to its ecological diversity and complex host–environment interfaces. This study explored the genetic diversity and structure of circulating pathogenic *Leptospira* spp. in Brazil through a single-locus sequence typing (SLST) analysis based on the *secY* gene. A total of 531 sequences were retrieved from GenBank and subjected to phylogenetic and haplotype diversity analyses. Maximum likelihood reconstruction revealed strongly supported clades for seven species, with *L. interrogans* being the most prevalent and broadly distributed across hosts and regions. This species showed evidence of clonal expansion, with a dominant haplotype (*n* = 242) shared by humans, domestic animals, and wildlife. In contrast, *L. santarosai* and *L. noguchii* exhibited high haplotypic diversity and reticulated network structures, reflecting greater evolutionary variability. The species *L. kirschneri* and *L. borgpetersenii* displayed reduced haplotypic variation, the latter mainly associated with cattle, consistent with its host-adapted profile. Host- and biome-based haplotype networks revealed both the broad ecological adaptability of certain lineages and the exclusive presence of haplotypes restricted to specific environments, such as those found in marine mammals from the Atlantic Ocean. Genetic distance analyses confirmed the strong taxonomic resolution of the gene *secY*, which effectively distinguished closely related species while capturing intraspecific diversity. These findings provide a comprehensive molecular overview of pathogenic *Leptospira* in Brazil, highlighting ecological connectivity across hosts and biomes, as well as the contrasting evolutionary dynamics among species. Beyond describing genetic patterns, our analyses emphasize evolutionary processes, host–environment connectivity, and the implications for One Health. This integrative framework strengthens the basis for surveillance and control strategies in other endemic regions in the world.

## 1. Introduction

Leptospirosis is a widespread zoonotic disease caused by pathogenic species of the genus *Leptospira*, which circulate among multiple animal hosts and persist in diverse ecological settings [1]. More than 160 mammalian species, along with several reptile and amphibian species, have been identified as hosts or reservoirs of the bacteria. These animals shed the pathogen primarily through urine, releasing it into the environment and contaminating soil and water sources [2]. The primary route of human infection is indirect, occurring when mucous membranes (eyes, mouth, nose) or abraded skin come into contact with water, soil, or food contaminated by the urine of infected animals. Risk is heightened in humid environments, during flooding, or in waterlogged soils, which enhance the survival and dissemination of leptospires, particularly in urban settings with inadequate sanitation and high rodent densities [3]. Intense rainfall events can further mobilize leptospires from soil into surface waters, thereby amplifying human exposure [4]. Given its complex transmission network involving humans, animals, and the environment, leptospirosis exemplifies a quintessential One Health challenge, demanding integrated surveillance and control strategies across sectors [5].

Globally, leptospirosis represents one of the most widespread zoonoses of bacterial origin, with an estimated one million human cases annually and mortality exceeding 60,000 deaths per year. The disease burden is especially high in tropical countries of South America and Southeast Asia, where climatic conditions favor environmental persistence and host density is elevated [6]. Brazil represents one of the most important global hotspots for leptospiral transmission due to its ecological heterogeneity and complex human–animal–environment interfaces. The country encompasses six major biomes, Amazon, Atlantic Forest, Cerrado, Caatinga, Pantanal, and Pampa, each harboring distinct climatic regimes, wildlife communities, and anthropogenic pressures. This environmental mosaic provides ideal conditions for the coexistence of multiple *Leptospira* species maintained by both wild and domestic hosts [7]. In the country, human leptospirosis is endemic and often reaches epidemic levels during the rainy season, particularly in capital cities and metropolitan areas. Outbreaks are closely linked to flooding, inadequate waste management, and high rodent infestation, which together increase exposure in low-income communities. In rural settings, occupational contact with livestock, contaminated water and soil represent major risk factors [8]. Urban incidence is highest in the North and Northeast regions, whereas rural cases predominate in the Southeast and South [9].

*Leptospira* remains a taxonomically complex and genetically diverse genus, encompassing more than 60 species that display a continuum of pathogenicity and ecological adaptation [10]. Although several local studies have characterized *Leptospira* spp. from specific hosts or regions in Brazil, comprehensive nationwide assessments integrating phylogenetic, ecological, and host data are still scarce. Most available information is fragmented or limited to regional case studies, lacking a unified comparative framework across biomes. Such integrative molecular analyses are essential to elucidate transmission pathways, assess genetic variability within and between species, and understand the ecological connectivity of circulating lineages. In this context, single-locus sequence typing (SLST) has proven to be a particularly valuable approach, especially in situations where bacterial isolation is unfeasible and only clinical samples are available. This technique relies on a single, highly informative gene that provides sufficient resolution for both genetic and phylogenetic analyses and has already been successfully applied to other bacterial groups [11,12]. The *secY* gene has emerged as one of the most informative loci for molecular detection and classification of pathogenic *Leptospira*. This housekeeping gene, which encodes a component of the protein translocation channel, combines high interspecific variability with sufficient conservation to allow amplification from a wide range of clinical and environmental samples. Its sequence polymorphism enables both species-level identification and intraspecific differentiation, while maintaining strong phylogenetic congruence with multilocus typing and whole-genome approaches. Furthermore, the gene’s broad availability in public databases facilitates large-scale comparative analyses and epidemiological surveillance [13,14].

In this study, we investigated the phylogenetic diversity and haplotype structure of pathogenic *Leptospira* spp. in Brazil by analyzing sequences derived from human and animal sources. By combining phylogenetic reconstruction, haplotype network inference, and genetic distance analyses, we aimed to characterize species-level diversity, host and biome associations, and evolutionary patterns of the main pathogenic taxa circulating in the country. This integrative approach provides new insights into the molecular epidemiology of *Leptospira* in a megadiverse tropical country and establishes a framework for One Health surveillance and control strategies.

## 2. Materials and Methods

### 2.1. Data Collection and Curation

Nucleotide sequences were obtained from the NCBI GenBank repository using the query “Leptospira AND Brazil AND secY” (accessed on 15 July 2025). We included only sequences with confirmed species-level identification and available metadata regarding host/source and geographic origin. Entries described solely as “*Leptospira* sp.” without species assignment were discarded, since accurate species identification is essential for molecular epidemiology analyses [15]. For this study, only pathogenic taxa belonging to the P1 clade, as proposed [10], were retained, while intermediate and saprophytic species were excluded. Each selected record was manually checked and annotated with key attributes: GenBank accession, species name, strain or sample identifier, host species, location of identification, and bibliographic reference. The curated dataset was organized in an Excel spreadsheet.

To allow broader ecological and epidemiological interpretation, host species were grouped into seven general categories: “Domestic large land animals”, “Wild land animals”, “Pets”, “Humans”, “Marine mammals” and “Urban rodents”. Likewise, sampling sites were assigned to one of the five main Brazilian biomes defined by the Brazilian Institute of Geography and Statistics (IBGE): Amazon, Atlantic Rain Forest, Pampa, Cerrado (Savanah), and Caatinga (semi-arid), according to the original collection site. The Atlantic Ocean was treated as a separate category (coastal-marine system, CMS).

### 2.2. Phylogenetic Analysis

Only *secY* sequences longer than 400 base pairs were retained to ensure reliable alignments and robust phylogenetic inference, as shorter fragments may reduce tree resolution and inflate homoplasy. For genome-derived records, *secY* sequences were extracted using the NCBI Genome Annotation tool, based on the corresponding GenBank or BioProject accession numbers. All command-line analyses were performed using the WSL terminal environment to ensure reproducibility and compatibility with bioinformatics tools. All *secY* sequences corresponded to a partial fragment of the gene, covering approximately positions 250–655 of the reference strain *L. interrogans* Fiocruz L1-130 (GenBank accession AE016823). After alignment and trimming, the final analyzed fragment spanned 405 base pairs (bp).

Sequences were aligned using MAFFT v7.505 [16] with default settings. Phylogenetic reconstruction was performed using IQ-TREE v2.2.6 [17], applying the Tamura–Nei (TN93) substitution model, as it was the best-fitting DNA substitution model determined by the Bayesian information criterion, with 1.000 ultrafast bootstrap replicates to assess branch support. A bootstrap value of ≥70% was considered indicative of strong support. The maximum likelihood (ML) tree generated was subsequently imported into MEGA X v11.0.13 [18] for visualization and editing. Minor adjustments to tree layout, taxon labels, and branch display were made to improve clarity and produce the final version used for interpretation and figure export.

### 2.3. Haplotype Networks

To further explore genetic patterns, median-joining haplotype networks were constructed using PopArt v1.7 using the median-joining inference method [19], based on the aligned *secY* dataset for each *Leptospira* species. Two separate networks were generated: one grouping haplotypes by host category and another by biome (as previously defined). Annotation was performed via a custom NEXUS input file to assign colors and groupings.

For single-locus analyses such as this, identical *secY* sequences were grouped into unique haplotypes, defined as sets of identical nucleotide sequences within the alignment. The term “haplotype” is used here in its phylogenetic context to describe sequence clusters derived from a single genetic locus, rather than the multilocus “sequence types (STs)” used in conventional MLST schemes.

### 2.4. Estimation of Genetic Distances

To provide additional insights into the genetic cohesiveness of each species and the degree of divergence between taxa occurring in Brazil, intraspecific and interspecific genetic distances were calculated using MEGA X, based on the aligned *secY* sequences. Mean pairwise distances within and between species were calculated under the TN93 model. Standard error (SD) estimates were obtained by a bootstrap procedure (1000 replicates)

## 3. Results

### 3.1. Phylogenetic Relationships

**Figure 1:**
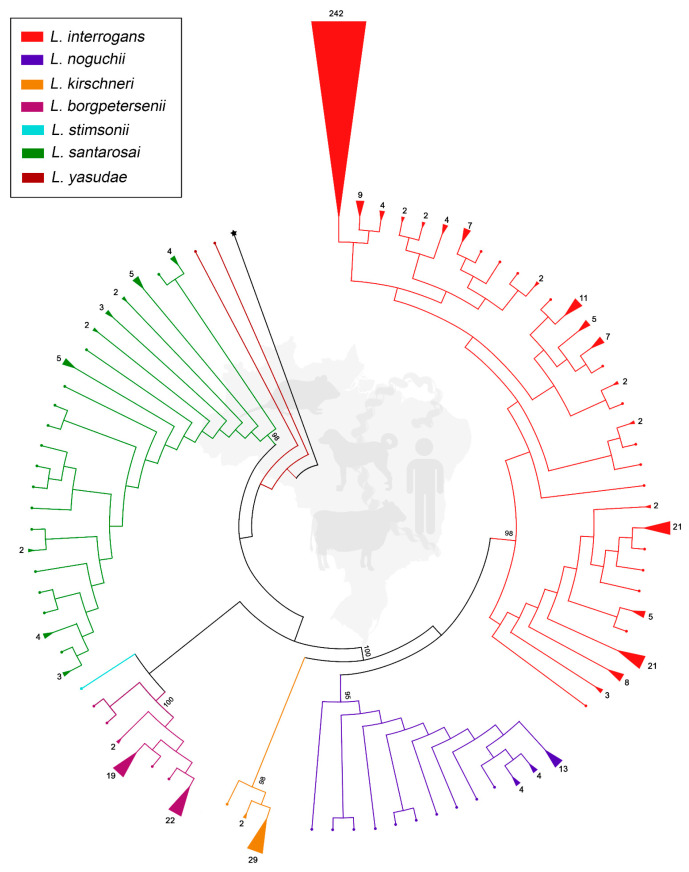
Maximum likelihood phylogenetic tree inferred from partial *secY* gene sequences (*n* = 531) of pathogenic *Leptospira* spp. identified in humans and animals in Brazil. Species identity is represented by colors, and triangle size is proportional to the number of identical sequences (number of sequences is shown). Small dots represent unique sequences. Numbers at nodes are bootstrap values. *Leptospira biflexa* strain Patoc is the outgroup taxon (represented by star).

A total of 531 sequences met the criteria and were included in the genetic analysis. Selected sequences and associated information are shown in Supplementary Material Table S1. The alignment spanned 405 bp, and the ML-TN93 tree recovered well-supported monophyletic clades for each recognized species, with bootstrap values exceeding 90% for most nodes. Notably, *L. interrogans* showed the highest number of total sequences and the greatest representation across multiple hosts and regions (Figure 1 and Supplementary Material Table S1). Moreover, it exhibited a remarkable clonal expansion represented by a predominant haplotype within the dataset comprising 242 sequences, including the reference strain Fiocruz L1-130. Other frequent haplotypes within *L. interrogans* also contained 21 and 11 sequences.

*Leptospira noguchii*, *L. kirschneri*, and *L. borgpetersenii* showed less diversity of haplotypes, with a predominance of one or two groups among 4 and 29 sequences, while *L. santarosai* presented several haplotypes formed by a maximum of 5 sequences, as well as presenting many unique sequences. The recently described species *L. yasudae* and *L. stimsonii* were represented by fewer sequences, but still formed distinct clusters, clearly indicating their genetic divergence from previously described species (Figure 1).

### 3.2. Haplotype Network Analysis by Host and Biome

**Figure 2:**
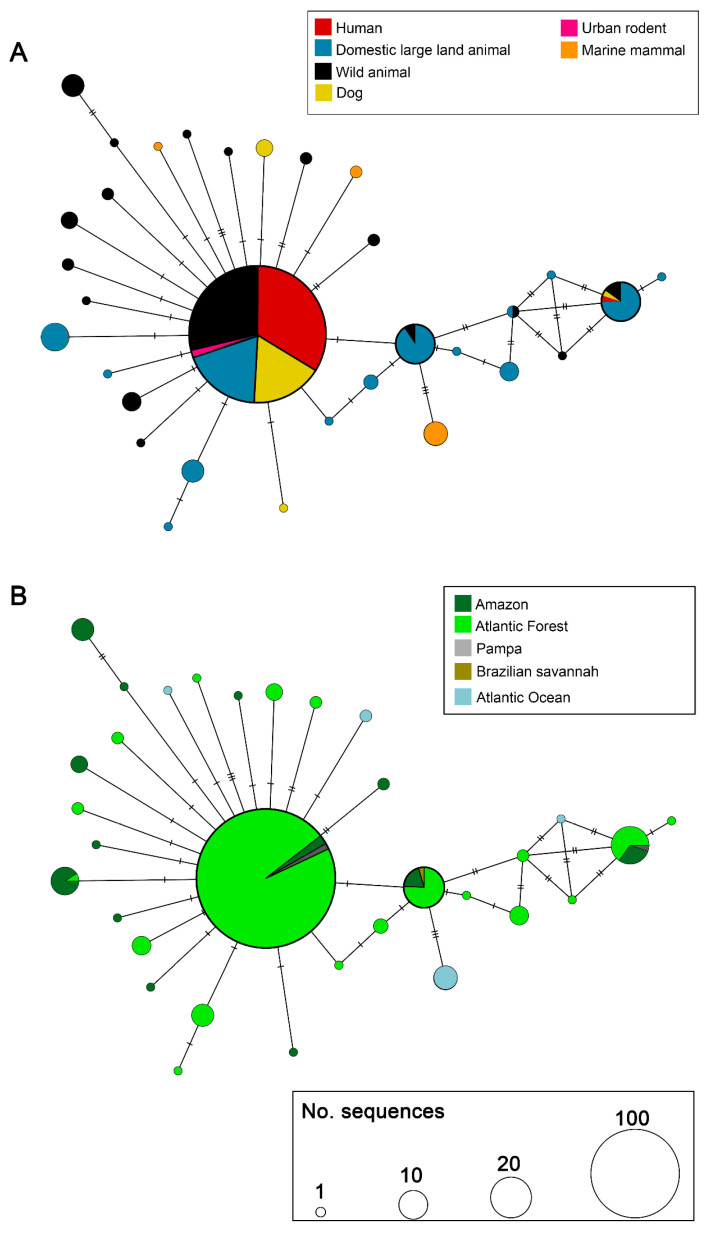
Haplotype network of *Leptospira interrogans* based on *secY* gene sequences. (**A**) Distribution of haplotypes according to host category. (**B**) Distribution of haplotypes according to the Brazilian biome. Each circle represents a unique haplotype, and its size is proportional to the number of sequences. Colors within circles indicate the host category (**A**) or biome (**B**), as specified in the legends. Hatch marks between circles represent the number of mutational steps between haplotypes.

The haplotype network based on *L. interrogans* sequences (Figure 2) revealed a total of 19 haplotypes, with one central haplotype comprising the majority of sequences (*n* = 242). This predominant haplotype (Fiocruz L1-130-like group) included representatives from several host categories, being practically equally distributed among humans, wild animals, domestic large land animals, and dogs, with few urban rodents. Other larger haplotypes, including about 20 sequences, are mostly from bovine origin. Small haplotypes, genetically close to the larger ones, were formed by sequences from marine mammals, dogs, and wild animals (Figure 2A).

In terms of geographic distribution (Figure 2B), the central haplotype encompassed sequences with absolute predominance of Atlantic Forest, with few from Amazon and Pampa. Several haplotypes were exclusive to the Atlantic Forest and Amazon. Sequences from the Atlantic Ocean formed three exclusive haplotypes, not shared with other biomes, such as that presented in the host network. The few sequences from Cerrado were identical to the sequences from Atlantic Forest and Amazon.

The sequences comprising each haplotype for the five species are detailed in Supplementary Material Table S2.

**Figure 3:**
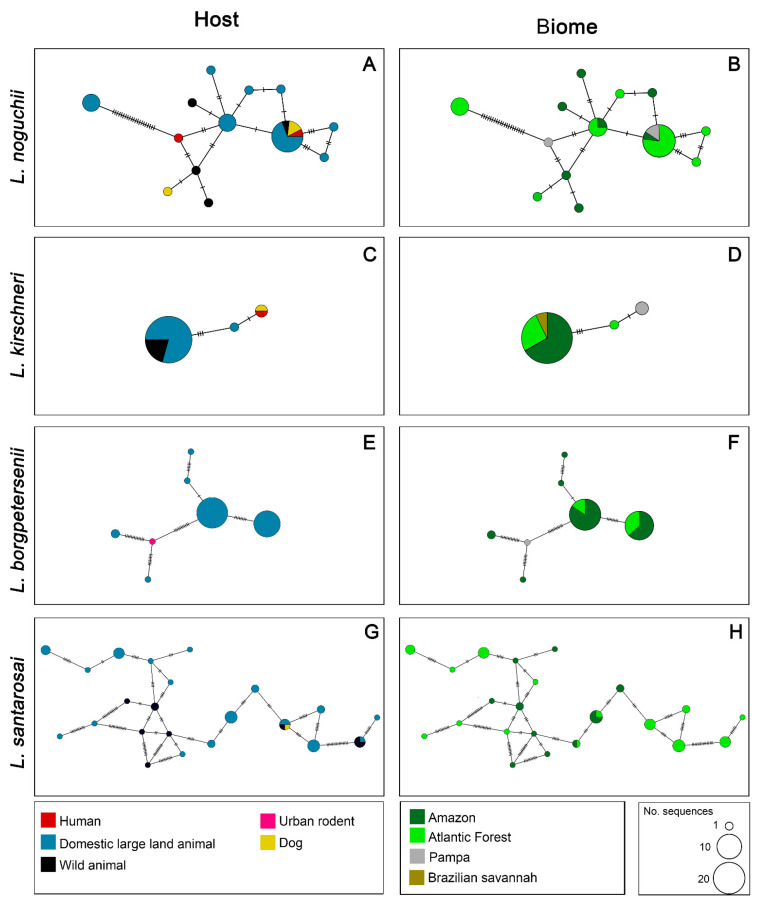
Haplotype networks of four pathogenic *Leptospira* species circulating in Brazil based on *secY* gene sequences. Each row represents one species: *L. noguchii, L. kirschneri, L. borgpetersenii,* and *L. santarosai*, respectively. The left column shows the distribution of haplotypes by host category (**A**,**C**,**E**,**G**), and the right column shows the distribution by biome (**B**,**D**,**F**,**H**). Each circle represents a unique haplotype, and its size is proportional to the number of sequences. Colors within circles correspond to the host categories (left) or biomes (right) as indicated in the legends. Hatch marks between circles represent the number of mutational steps separating haplotypes.

For *L. noguchii*, the haplotype network exhibited a high degree of haplotypic diversity, with several intermediate nodes and no single dominant haplotype. The sequences were broadly distributed among multiple haplotypes, many of which were shared across host categories. Domestic large land animals represented the majority of hosts, followed by wild animals, humans, and dogs (Figure 3A). In the biome-specific network, haplotypes were spread across three biomes, with notable abundance in the Atlantic Forest. Several haplotypes were exclusive to a single biome, while two were shared across more regions (Figure 3B).

For *L. kirschneri*, the network revealed low haplotypic diversity, with a predominant central haplotype encompassing most sequences. This main haplotype included sequences from domestic, large land and wild animals. A few peripheral haplotypes were connected by a few mutational steps (Figure 3C). In the biome-based network, sequences were mainly associated with the Atlantic Forest, Amazon, and Cerrado, with a small number linked to the Pampa biome, which formed an exclusive haplotype (Figure 3D).

*Leptospira borgpetersenii* also showed low haplotypic diversity, with a few dominant haplotypes. The host haplotype network was formed almost exclusively by sequences originating from cattle, with a single haplotype composed of an urban rodent sequence (Figure 3E). In the biome-based network, sequences were predominantly from the Amazon, followed by the Atlantic Forest. The urban rodent sequence was from the Pampa biome (Figure 3F). Overall, the network structure was simpler compared to other species, with fewer mutational steps and a more clustered organization.

The network for *L. santarosai* revealed the highest haplotypic diversity among the species analyzed, with numerous haplotypes distributed along a reticulated and complex structure. This network structure was characterized by multiple internal connections, and no single haplotype was dominant. Host distribution showed dominance of domestic large land animals and wild animals, with smaller contributions from dogs (Figure 3G). The biome-based network was similarly diverse, with sequences from only two biomes, the Atlantic Forest and Amazon, with only two shared between regions (Figure 3H).

### 3.3. Genetic Distances Within and Between Leptospira Species

Genetic distances (TN93) based on 531 nucleotide sequences of the *secY* gene revealed low to moderate intraspecific variability across the five pathogenic *Leptospira* species circulating in Brazil (bold values along the diagonal in Table 1). The lowest intraspecific distance was observed for *L. kirschneri* (0.002), followed by *L. noguchii* (0.017) and *L. interrogans* (0.004), while *L. santarosai* exhibited the highest intraspecific divergence (0.034). Regarding interspecific comparisons (below the diagonal in Table 1), the smallest genetic distances were found between *L. interrogans*, *L. noguchii*, and *L. kirschneri* (ranging from 0.02 to 0.03), suggesting closer evolutionary relationships among these taxa. In contrast, *L. borgpetersenii* and *L. santarosai* showed greater divergence in relation to the other species, with distances reaching up to 0.24 when compared to *L. noguchii*. Standard deviations were generally low, indicating consistency in pairwise comparisons (above diagonal in italics in Table 1).

## 4. Discussion

To explore the genetic structure of circulating *Leptospira* spp. in Brazil, 531 *secY* gene sequences were selected, a marker well established for both taxonomic and epidemiological resolution [13,14]. This extensive dataset, encompassing sequences from multiple hosts and biomes, provided a robust basis for investigating lineage diversification and geographic structuring at a national scale. Herein, the *secY* locus has been consistently shown to balance interspecific conservation with sufficient intraspecific variability, allowing the reconstruction of reliable phylogenetic relationships while detecting microevolutionary signals within populations. By combining *secY*-based phylogenetic reconstruction, haplotype network analysis, and genetic distance estimation, we were able to delineate distinct evolutionary clusters corresponding to recognized species and to visualize the degree of haplotypic connectivity among hosts and regions.

The maximum likelihood phylogeny recovered strongly supported monophyletic clades for each species, with *L. interrogans* showing the largest number of sequences and broadest distribution across hosts and regions. This dominance underscores the remarkable ecological versatility of *L. interrogans*, a species capable of maintaining stable transmission cycles in both urban and rural settings, facilitated by its adaptability to a wide range of mammalian hosts [20]. A striking feature was the evidence of clonal expansion, with a single haplotype encompassing 242 sequences, including the reference strain Fiocruz L1-130, detected in humans, domestic animals, and wildlife, and spanning multiple biomes. Such extensive representation across hosts and regions indicates that this clone has achieved ecological dominance, likely supported by its capacity to persist in both maintenance and incidental hosts. The presence of identical sequences in geographically distant areas also suggests recent or ongoing gene flow among populations, possibly mediated by the movement of livestock, companion animals, and synanthropic rodents. The success of this lineage reflects both low genetic variability and wide ecological adaptability, consistent with its role as the major cause of human outbreaks in Brazil, especially in urban centers with high rodent infestation [21]. This evolutionary success likely results from a combination of host plasticity and environmental resilience, allowing the bacteria to survive under fluctuating conditions of humidity, temperature, and salinity. The lineage’s widespread distribution also suggests a long-term evolutionary stability, possibly linked to efficient maintenance within rodent reservoirs and recurrent spillovers to other mammals. The persistence of this haplotype across hosts and landscapes highlights its epidemiological significance: clonal stability may facilitate efficient transmission, while reduced antigenic diversity could simultaneously favor vaccine targeting and amplify risks of large-scale outbreaks if this lineage continues to expand unchecked. From an epidemiological standpoint, this pattern illustrates the dual nature of clonal expansion: it enhances predictability for diagnostic and vaccine development yet increases vulnerability to epidemic resurgence when environmental or climatic conditions become favorable for transmission. Continuous genomic monitoring of this dominant haplotype could therefore serve as an early-warning indicator for urban leptospirosis outbreaks. A clonal subpopulation of *L. interrogans* was previously reported during leptospirosis outbreaks in Brazil [22]. Similarly, clonal expansion of *L. interrogans* was identified as the predominant driver of leptospirosis cases in the Indian archipelagos [23]. These findings, observed across independent epidemiological settings, highlight a possible broader global trend in which a few highly adapted *L. interrogans* lineages sustain the majority of human infections. Such convergence across continents suggests evolutionary optimization toward transmission efficiency rather than diversification. However, further studies employing standardized genetic characterization approaches are needed to confirm this trend and elucidate its evolutionary and epidemiological implications.

By contrast, *L. kirschneri* and *L. borgpetersenii* presented simpler network topologies, with limited haplotypic variation and few mutational steps between clusters. The predominance of a small number of haplotypes may reflect host adaptation and reduced environmental survival, particularly in the case of *L. borgpetersenii*, which has undergone genome reduction and relies more on direct host-to-host transmission [24,25]. Recent genomic studies reinforce this restricted variability, showing that *L. borgpetersenii* lineages circulating in cattle often cluster into very few haplotypes or STs, highlighting clonal expansion and limited environmental persistence [26,27]. Similarly, *L. kirschneri* isolates frequently display low genetic variability and well-structured clusters, consistent with local host adaptation and geographically restricted lineages [28]. This restricted diversity is consistent with their frequent association with cattle and other livestock, reinforcing the role of domestic animals in maintaining adapted *Leptospira* lineages and shaping transmission cycles [29].

Haplotype networks stratified by host and biome provided further resolution of phylogeographic patterns. For *L. interrogans*, the dominant haplotype was widely shared among humans, cattle, dogs, and wildlife, and was particularly prevalent in the Atlantic Forest biome. Such patterns corroborate previous findings of clonally dominant lineages circulating in small mammals in this biome [30] and illustrate strong ecological connectivity across hosts and environments. Shared haplotypes spanning different Brazilian biomes emphasize lineage stability at continental scales, suggesting successful dispersal mechanisms possibly mediated by synanthropic reservoirs such as rodents. Similar patterns have been documented elsewhere: in urban areas of Guangzhou, China, the coexistence of *L. interrogans*, *L. borgpetersenii*, and *L. kirschneri* in commensal rodents was reported, highlighting their role as long-term carriers and effective vehicles for dissemination across ecological boundaries [31]. At the genomic level, Chinchilla and colleagues (2023) analyzed 914 genomes of *L. santarosai* from the Americas and revealed both region-specific clonal groups and evidence of historical lineage movements across distant areas, pointing to global-scale dispersal dynamics. Together, these findings suggest that haplotype sharing in Brazil may not be an isolated phenomenon but rather part of a broader ecological and evolutionary pattern in which synanthropic reservoirs facilitate the persistence and geographic expansion of pathogenic *Leptospira* worldwide.

Exclusive haplotypes identified in marine mammals from the Atlantic Ocean expand the ecological boundaries of leptospiral circulation. Although the genetic distances separating these lineages were minimal (one to three substitutions), their exclusivity suggests ecological isolation, with limited connectivity to terrestrial reservoirs. Nevertheless, the zoonotic potential of this environment cannot be disregarded, as marine–terrestrial interfaces may represent overlooked transmission routes [32]. These findings reinforce the need for frameworks that encompass both terrestrial and aquatic ecosystems..

The analysis of genetic distances supported the phylogenetic and haplotype network results. Intraspecific distances were lowest for *L. kirschneri* (0.002) and *L. interrogans* (0.004), consistent with clonal expansion and host-adapted transmission. By contrast, *L. santarosai* showed the highest intraspecific divergence (0.034), reinforcing its evolutionary variability. Interspecific distances indicated closer genetic relatedness among *L. interrogans*, *L. noguchii*, and *L. kirschneri*, while *L. borgpetersenii* and *L. santarosai* were more distinct, a finding consistent with whole-genome and cgMLST analyses [10,33,34]. Importantly, the absence of overlap between maximum intraspecific and minimum interspecific distances underscores the strong taxonomic resolution of *secY*, supporting its use as a robust marker for species delimitation and intraspecific diversity assessment.

From a One Health perspective, the genetic connectivity observed among *Leptospira* lineages circulating in humans, domestic animals, and wildlife underscores the importance of coordinated surveillance across sectors. Molecular approaches such as *secY*-based typing provide a practical and cost-effective tool to monitor pathogen flow between hosts and environments, identify potential spillover interfaces, and guide preventive measures adapted to local ecological contexts. By bridging molecular epidemiology with ecological data, this study contributes to the integrated understanding required for sustainable leptospirosis control in endemic tropical regions.

Despite these advances, some limitations should be considered. The reliance on a single-locus marker, although informative, precludes the detection of recombination events and fine-scale genomic adaptations. Whole-genome approaches will be essential to uncover hidden diversity and clarify evolutionary processes such as horizontal gene transfer. Furthermore, sequence availability is uneven across hosts and regions, potentially biasing haplotype frequencies and underrepresenting circulation in poorly sampled biomes such as the Caatinga and Pantanal. Because the dataset analyzed here was entirely derived from publicly available *secY* sequences, it reflects the representativeness of deposited records rather than the true prevalence of genotypes in nature. The apparent predominance of certain haplotypes may therefore result from uneven research efforts, regional sampling preferences, or repeated sequencing of epidemiologically important isolates. Consequently, the patterns described in this study should be interpreted as indicators of genetic diversity and connectivity within the available dataset, not as estimates of infection frequency or epidemiological dominance. Finally, future studies combining Brazilian and international datasets will be essential to determine whether the country harbors unique endemic clades or mainly reflects the global diversity structure of pathogenic *Leptospira*. In this context, formal nomenclature of *secY*-based haplotypes should be pursued to facilitate comparability among research groups and ensure consistent lineage recognition across regions worldwide. Addressing these gaps through coordinated sampling efforts will be critical for a truly comprehensive picture of *Leptospira* diversity in Brazil and in the world.

## 5. Conclusions

This study provides a comprehensive molecular overview of the genetic diversity of pathogenic *Leptospira* circulating in Brazil. Phylogenetic analyses based on the *secY* gene revealed well-supported clades for the main pathogenic species, while haplotype network analyses uncovered contrasting evolutionary patterns, ranging from clonal expansion in *L. interrogans* to extensive haplotypic diversity in *L. santarosai* and *L. noguchii*. Host and biome-based networks highlighted both the ecological adaptability of dominant lineages and the existence of exclusive haplotypes in specific environments, such as marine mammals in the Atlantic Ocean. Genetic distance estimates confirmed the strong taxonomic resolution of *secY*, distinguishing closely related species while capturing intraspecific diversity. Together, these findings confirm the complex interplay of host, environment, and pathogens shaping leptospiral diversity. By bridging phylogenetics with ecological and epidemiological contexts, this work contributes to a deeper understanding of leptospiral transmission dynamics and provides a valuable framework for One Health surveillance and control strategies in endemic regions.

## Figures and Tables

**Table 1:** Interspecific genetic distance (below the diagonal) between the five pathogenic *Leptospira* species circulating in Brazil based on 534 nucleotide sequences of the *secY* gene. Standard deviation is shown above the diagonal in italics. Intraspecific genetic distances are in bold along the diagonal.

	*L. interrogans*	*L. noguchii*	*L. kirschneri*	*L. borgpetersenii*	*L. santarosai*
*L. interrogans*	**0.004**	*0.02*	*0.02*	*0.03*	*0.03*
*L. noguchii*	0.11	**0.017**	*0.02*	*0.03*	*0.02*
*L. kirschneri*	0.11	0.11	**0.002**	*0.03*	*0.02*
*L. borgpetersenii*	0.23	0.21	0.21	**0.015**	*0.02*
*L. santarosai*	0.23	0.24	0.20	0.11	**0.034**

## Data Availability

The data presented in this study are openly available in GenBank at https://www.ncbi.nlm.nih.gov/genbank/ (accessed on 30 October 2025).

## Supplementary Materials

The following supporting information can be downloaded at: https://www.mdpi.com/article/10.3390/microorganisms13112512/s1, Table S1: Complete curated dataset of the 531 pathogenic *Leptospira* sequences included in this study. For each record, the table compiles genetic, geographic, and host-related metadata retrieved from GenBank; Table S2: List of sequences included in each haplotype for the five pathogenic *Leptospira* species analyzed in this study. One species per worksheet is shown. For each species, haplotypes were defined based on *secY* gene sequences (>400 bp) through haplotype network analysis on PopArt software.
